# Two types of potential functions and their use in the modeling of information: two applications from the social sciences

**DOI:** 10.3389/fpsyg.2015.01513

**Published:** 2015-10-21

**Authors:** Emmanuel E. Haven

**Affiliations:** School of Management, Institute for Quantum Social and Cognitive Science and Institute of Finance, University of LeicesterLeicester, UK

**Keywords:** potential functions, quantum mechanics

## Abstract

In this paper we consider how two types of potential functions, the real and quantum potential can be shown to be of use in a social science context. The real potential function is a key ingredient in the Hamiltonian framework used in both classical and quantum mechanics. The quantum potential however emerges in a different way in quantum mechanics. In this paper we consider both potentials and we attempt to give them a social science interpretation within the setting of two applications.

## 1. Introduction

Potential functions are to physicists what utility functions are to economists: they are both examples of fundamental workhorse tools. But can there exist some connection? Utility functions, *u*, are defined as: *u*:*C* → ℝ, where *C* is a set of objects. A preference relation on two objects, *x* and *y* such that, say, *x*≻*y* will imply that *u*(*x*)>*u*(*y*) under the necessary conditions that the preference relation is transitive and asymmetric. This paper will not pretend to be at a level of rigor which has been attained in economics based preference theory. Examples of such rigor abound in the various expected utility frameworks, and some papers in this special issue will be devoted to probing how deviations from central axioms like the sure-thing principle can be explained with the aid of quantum structures. Our objective in this paper is modest: we would like to inform (and maybe convince) the reader that with the help of two types of potential functions we can model, in a reasonably successful way, information. This information can include parameters which refer to attitudes toward risk (preferences for risk).

In the next section we introduce the basic structure where those two potential functions can exist. In the next two sections we consider two applications from the social sciences which will attempt to highlight the possible added value of using those potential functions in a non-physics setting. We also consider in the last section of the paper a brief discussion on the relevance of such potential functions in real world market settings.

## 2. Basic structure where two potential functions can occur

The so called stochastic equivalent of the Hamilton-Jacobi equations provides for at least, according to the author of this paper, a sort of twilight state where we move from classical mechanics to stochastics and then to quantum mechanics. The hydrodynamic approach to quantum mechanics was developed by Edward Nelson, and in this section we will provide for the essentials of the basic structure which we need, to develop the examples in Sections 3 and 4. We use elements from the set up in the paper by Haven (2015). For a lot more detail, the paper by Nelson (1966) is the essential reference. The book by Paul and Baschnagel (1999) is also an excellent source (see also Haven and Khrennikov, 2013).

We will follow, as in the paper by Haven (2015) and Paul and Baschnagel (1999) approach to Nelson's theory. We also use the same notation. Consider a position in space, *x*, indexed by time (which we denote here by the index *n***)**. Let time contract to zero, one can then write as in Nelson (1966) and Paul and Baschnagel (1999), that $\frac{dx_{+}}{dt}≃\frac{x_{n+1}-x_{n}}{\epsilon }$ and $\frac{dx_{-}}{dt}≃\frac{x_{n}-x_{n-1}}{\epsilon }$, where ϵ denotes the difference in time *t*, and *d* indicates the infinitesimal differential operator. In the area of finance, the so called Brownian motion is a very common way of describing the random evolution of an asset price over time. Brownian motions are well-known in physics, especially with the formalization Einstein gave of such motions. As we mention in Haven (2015), Nelson (1966), and Paul and Baschnagel (1999), define two Brownian motions of the following types:

(1) $$\begin{array}{l} dx\left(t\right)=b_{+}\left(x,t\right)dt+\sigma dW\left(t\right); \end{array}$$

where *dW*(*t*) is a Wiener process; σ is the diffusion coefficient and *b*_+_(*x, t*) is the so called drift function. They also define:

(2) $$\begin{array}{l} dx\left(t\right)=b_{-}\left(x,t\right)dt+\sigma dW\left(t\right). \end{array}$$

What is now the difference between the two drift functions? Still following the set up in Haven (2015), Nelson (1966), and Paul and Baschnagel (1999) define:

(3) $$\begin{array}{l} D_{+}x\left(t\right)=\underset{\epsilon \to 0}{\lim}E\left[\frac{x_{n+1}-x_{n}}{\epsilon }\right]=b_{+}\left(x,t\right), \end{array}$$

and also:

(4) $$\begin{array}{l} D_{-}x\left(t\right)=\underset{\epsilon \to 0}{\lim}E\left[\frac{x_{n}-x_{n-1}}{\epsilon }\right]=b_{-}\left(x,t\right). \end{array}$$

Note that *E* is the expectation operator. This paper has mentioned in its introduction that we consider two types of potentials. But what are they? The real potential is the first type, well-known from elementary classical mechanics. The real potential formalizes potential energy. The second type, is the so called quantum potential which emerges from inserting the polar form of a wave function into the Schrödinger partial differential equation. The uses of those potentials were first proposed by Khrennikov (1999) already more than 10 years ago. Haven (2015)^1^, indicates that a key component of the so called quantum potential, can be written as follows:

(5) $$\begin{array}{rcl} \frac{\nabla^{2}R^{\prime }}{R^{\prime }} & = & \frac{1}{\sigma^{2}}\frac{\partial }{\partial x}\left[\frac{1}{2}\left(b_{+}\left(x,t\right)-b_{-}\left(x,t\right)\right)\right]\\ +\frac{1}{4\sigma^{4}}\left[b_{+}{\left(x,t\right)}^{2}-2b_{+}\left(x,t\right)b_{-}\left(x,t\right)+b_{-}{\left(x,t\right)}^{2}\right], \end{array}$$

where *R*′ is a scalar field. We then argue in Haven (2015)^2^ that if we set *b*_+_(*x, t*) = *b* and *b*_−_(*x, t*) = *c* where *b*≠*c*; *b*, *c*∈ℝ:

(6) $$\begin{array}{l} \frac{\nabla^{2}R^{\prime }}{R^{\prime }}=\frac{1}{4\sigma^{4}}\left[b^{2}-2bc+c^{2}\right]=\frac{1}{4\sigma^{4}}{\left(b-c\right)}^{2}. \end{array}$$

If one consider the classical mechanical equivalent of the quantum potential, one multiplies $\frac{\nabla^{2}R^{\prime }}{R^{\prime }}$ with $\frac{-m\sigma^{4}}{2}$, where *m* is mass. We note that use is made of the conversion: $\sigma^{2}=\frac{\hbar }{m}$. We note that such conversion requires further discussion (see Nelson, 1985). Hence, we obtain, as in Haven (2015)^3^:

(7) $$\begin{array}{l} \frac{-m\sigma^{4}}{2}\frac{\nabla^{2}R^{\prime }}{R^{\prime }}=\frac{-m}{8}{\left(b-c\right)}^{2}. \end{array}$$

We are now ready to consider our first application.

## 3. Application 1: three examples showing which additional information is brought on by the use of the quantum potential

The Newtonian motion with both the real and quantum potentials is: *m*.*a* = −∇(*V*+*Q*) (see for instance Haven and Khrennikov, 2013, for more detail). If one consider the force, −∇*Q*, to be applied on Equation (7), one can see immediately that:

(8) $$\begin{array}{l} -\nabla Q=-\frac{\partial }{\partial x}\left[\frac{-m\sigma^{4}}{2}\frac{\nabla^{2}R^{\prime }}{R^{\prime }}\right]=\frac{-\partial }{\partial x}\left[\frac{-m}{8}{\left(b-c\right)}^{2}\right]=0. \end{array}$$

The force derived from the real potential, −∇*V*, is −*b*_+_(*x, t*) or −*b*_−_(*x, t*). From an economics point of view, such force can be interpreted as an expected return. Hence, this force can thus incorporate preferences for risk.

In order to give an interpretation to the quantum potential, we need to re-consider Equation (5) but now for the case where *b*_+_(*x, t*) and/or *b*_−_(*x, t*) are not constant. Hence, let us consider the simple case where *b*_+_(*x, t*) = μ*x*, where we can set that μ is now the expected return. We note that making μ to be such an expected return follows in parallel to what is done in financial economics, where the drift term of the geometric Brownian motion is a product of the expected return and the position variable (i.e., the value of the stock price for instance). Let us assume, for easiness of purpose, that *b*_−_(*x, t*) = 0. In this case, we are *not* in Newtonian mechanics since we are now explicitly imposing that *b*_+_(*x, t*)≠*b*_−_(*x, t*). We re-consider Equation (5) again:

(9) $$\begin{array}{l} \frac{\nabla^{2}R^{\prime }}{R^{\prime }}=\frac{1}{\sigma^{2}}\frac{\partial }{\partial x}\left[\frac{1}{2}\left(\mu x\right)\right]+\frac{1}{4\sigma^{4}}\left[\mu^{2}x^{2}\right]. \end{array}$$

The quantum potential, *Q*, is written as: $\frac{-m\sigma^{4}}{2}\frac{\nabla^{2}R^{\prime }}{R^{\prime }}=\frac{-m\sigma^{2}}{2}\frac{\partial }{\partial x}\left[\frac{1}{2}\left(\mu x\right)\right]-\frac{m}{8}\left[\mu^{2}x^{2}\right].$ The force is then:

(10) $$\begin{array}{l} -\nabla Q=-\frac{\partial }{\partial x}\left[\frac{-m\sigma^{2}}{2}\frac{\partial }{\partial x}\left[\frac{1}{2}\left(\mu x\right)\right]-\frac{m}{8}\left[\mu^{2}x^{2}\right]\right]. \end{array}$$

This yields then:

(11) $$\begin{array}{l} -\nabla Q=\frac{m}{4}\mu^{2}x. \end{array}$$

If we assume that the force on the real potential, −∇*V* = −*b*_+_(*x, t*) = −μ*x*, then, one can also write that:

(12) $$\begin{array}{l} -\nabla V-\nabla Q=-\mu x+\frac{m}{4}\mu^{2}x \end{array}$$

We observe from the above, that besides the information received, via the force on the real potential, i.e., the expected return times the position, additional information is now injected via the force on the quantum potential.

If we were to let $b_{+}\left(x,t\right)=\mu x^{2}$, then using Equation (5):

(13) $$\begin{array}{l} \frac{\nabla^{2}R^{\prime }}{R^{\prime }}=\frac{1}{\sigma^{2}}\frac{\partial }{\partial x}\left[\frac{1}{2}\left(\mu x^{2}\right)\right]+\frac{1}{4\sigma^{4}}\left[\mu^{2}x^{4}\right]. \end{array}$$

Using $\frac{-m\sigma^{4}}{2}\frac{\nabla^{2}R^{\prime }}{R^{\prime }}$ then the force delivered by both the quantum and real potentials (assuming $-\nabla V=-b_{+}(x,t)=-\mu x^{2})$ is:

(14) $$\begin{array}{l} -\nabla V-\nabla Q=-\mu x^{2}+\frac{m\mu^{2}}{2}x^{3}+\frac{m\sigma^{2}}{2}\mu . \end{array}$$

If we would set *b*_+_(*x, t*) = μ, then [see Equation (8)], we would only be able to write that:

(15) $$\begin{array}{l} -\nabla V-\nabla Q=-\mu \end{array}$$

Let us compare those simple cases, Equations (12, 14 and 15). We can observe that additional terms are added to the force on the real potential. Remark however that we have assumed that the drift term in the pure Newtonian environment, is the same as the drift terms *b*_+_(*x, t*) and *b*_−_(*x, t*). If we translate the pure Newtonian environment, into the Nelson framework we obtain $\nabla R\left(x,t\right)=\frac{E\left[\frac{dx_{+}}{dt}-\frac{dx_{-}}{dt}\right]}{2\sigma^{2}}=0$ and therefore, in that setting, *R*(*x, t*) is constant. This would mean that the density function, in the Nelson framework *e*^2*R*(*x, t*)^ would be constant. The quantum potential would also be zero. Hence, the equivalent information of the pure Newtonian setting into a Nelsonian setting would be senseless. However, the quantum potential still is comparable to the real potential, after all one can write: −∇*V*−∇*Q* = *m*.*a*! This is in some sense a dilemma.

Consider again Equations (12, 14 and 15), and let us re-write slightly, as follows:

- Under the Newtonian based theory if the expected return (i.e., force on real potential) is the expected return μ then the additional information (brought by the gradient of the quantum potential) is nil
- Under the Newtonian based theory if the expected return (i.e., force on real potential) is the expected return μ*x* then the additional information (brought by the gradient of the quantum potential) is [set *m* = 1 (please see below for a comment on such setting)]: $\frac{1}{4}\mu^{2}x$
- Under the Newtonian based theory if the expected return (i.e., force on real potential) is the expected return μ*x*^2^ then the additional information (brought by the gradient of the quantum potential) is [set *m* = 1 (please see below for a comment on such setting)]: $\frac{\sigma^{2}\mu }{2}+\frac{\mu^{2}}{2}x^{3}$

Given the reasonableness of this force on the quantum potential to exist (i.e., volatility is not zero and the uncertainty given by $\left(\frac{x_{n+1}-x_{n}}{\epsilon }\right)\neq \left(\frac{x_{n}-x_{n-1}}{\epsilon }\right)$ to exist, it would then be reasonable to claim that if the expected drift is given as μ*x* in a Newtonian world [where $\left(\frac{x_{n+1}-x_{n}}{\epsilon }\right)=\left(\frac{x_{n}-x_{n-1}}{\epsilon }\right)$ and σ = 0] then in a Nelson world this information would need to be augmented with: $\frac{1}{4}\mu^{2}x$. Similarly, if the expected drift is given as μ*x*^2^ in a Newtonian world [where $\left(\frac{x_{n+1}-x_{n}}{\epsilon }\right)=\left(\frac{x_{n}-x_{n-1}}{\epsilon }\right)$ and σ = 0] then in a Nelson world this information would need to be augmented with: $\frac{\sigma^{2}\mu }{2}+\frac{\mu^{2}}{2}x^{3}$.

Remark one very important issue which refers to the setting of *m* = 1 in the above discussion. The Nelson theory allows for a transition from pure Newtonian mechanics, into a stochastic environment [with the use of *R*(*x, t*) as a scalar field] and from there, into a further transition into quantum mechanics [with the use of *R*(*x, t*) now as an input into the wave function]. This transition from stochastics to quantum mechanics, also goes via the setting of $\sigma^{2}=\frac{\hbar }{m}$.

For a given finite σ^2^, in a quantum mechanical context when ℏ = σ^2^*m*, it would mean that *m* should be extremely!!! small indeed. The question becomes, whether the level of *m* has a continuum of values when transiting from the stochastic environment toward the quantum mechanics environment. If one considers the case $-\mu x+\frac{m}{4}\mu^{2}x$ then when *m* is extremely small, the term $\frac{m}{4}\mu^{2}x$ would need to be extremely small. The same can be said for: $-\mu x^{2}+\frac{m\mu^{2}}{2}x^{3}+\frac{m\sigma^{2}}{2}\mu$, where the terms which are added to μ*x*^2^ are then small too, because of small $m:\frac{m\mu^{2}}{2}x^{3}+\frac{m\sigma^{2}}{2}\mu$. It would be a major achievement, if indeed we could find how *m* behaves when transiting from Newtonian → stochastics (with *R* as scalar field) → quantum mechanics (with *R* as an input to the wave function).

## 4. Application 2. an example of how the real and quantum potentials can be used in lux's noise trader infection model

The “noise trader/infection” model was developed by Lux (1997) and we use it here to highlight the applications we can make, in a financial economics framework, of the potentials we have treated in our paper. From Equation (5), we can observe that σ^2^ is essential to define the quantum potential. We will make the simple assumption, for the purposes of the model treated here, that $E\left(\frac{dx_{-}}{dt}\right)=0$. There is a non-zero uncertainty due to the fact that $E\left(\frac{dx_{+}}{dt}\right)\neq E\left(\frac{dx_{-}}{dt}\right)$.

### 4.1. Brief set up of the lux model

The model has two main types of traders, fundamental and chartist (or also called noise) traders. The total number of chartist traders is 2*N*. They are divided into two subgroups, *n*_+_ (the number of noise traders who are positive about the development of the market) and *n*_−_ (the number of noise traders who are negative about the development of the market) and hence *n*_+_+*n*_−_ = 2*N*. An opinion index, which is, in the words of Lux (1997) (p. 8) “the distribution of attitudes among the ‘population’” is also constructed and it is defined as: $x=\frac{n_{+}-n_{-}}{2N}$. Remark that in the model, chartist traders may change from one subgroup to the other. The smallest difference in the opinion index is given by $\pm \frac{1}{N}$ and the asset price's minimal change is ± one cent. The central equation in which we are interested in for the purposes of our paper, is as follows (Equation 3.7b in Lux, 1997):

(16) $$\begin{array}{l} \frac{d\bar{p}}{dt}=\beta \left(\bar{x}T_{c}+T_{f}\left(p_{f}-\bar{p}\right)\right); \end{array}$$

where β is defined as (Lux, 1997, p. 8): “a parameter for the average speed of price adjustment in the presence of excess demand.”; $\bar{x}$ is the average distribution of attitudes; *T*_*c*_ measures the trading volume of the chartist traders; *T*_*f*_ measures the trading volume of the fundamental traders; *p*_*f*_ is the perceived fundamental value of the asset and $\bar{p}$ is the expected price.

### 4.2. Embedding lux's model in the quantum/real potential environment

If we want to embed the above model in the quantum/real potential model presented here in this paper, then a departure of Lux's model could be as follows:

(17) $$\begin{array}{l} d\bar{p}=\beta \left(\bar{x}T_{c}+T_{f}\left(p_{f}-\bar{p}\right)\right)dt+dW; \end{array}$$

where *dW* is a Wiener process as defined before. Embedding this departure of Lux's model in the quantum/real potential model, we can then write that:

(18) $$\begin{array}{l} E\left[\frac{d\bar{p}}{dt}\right]=\bar{x}\beta T_{c}+\beta T_{f}p_{f}-\bar{p}\beta T_{f}+E\left(dW\right); \end{array}$$

which can then be re-written as, using *E*(*dW*) = 0:

(19) $$\begin{array}{l} E\left[\frac{d\bar{p}}{dt}\right]=\bar{x}\beta T_{c}+\beta T_{f}p_{f}-\bar{p}\beta T_{f}. \end{array}$$

Remark that the $\bar{x}$ parameter could be interpreted as being closely linked to some implicit preference functional, which in this model, is driven by chartists. We remark that Equation (3.7a; p. 13) in Lux's paper Lux (1997) provides for the time dependent evolution of the $\bar{x}$ parameter.

### 4.3. Consequence of the absence of expectation operators

Remark that if we were to write Equation (16) (as it is thus written in Lux's model), as a result of having it embedded in our quantum/real potential model (thus now without expectation operator) then this would imply that $\frac{dW}{dt}=0$. If we now go back to the importance of expectation operators in Nelson's theory we can say the following. Imagine we were to not use such an operator on $\frac{dW}{dt}$. For instance, can we then still write that $\frac{dx_{+}}{dt}=b_{+}\left(x,t\right)$, using the Brownian motion Equation (1)? The answer to this question would impose the requirement that no time reversibility can exist. The argument is quite straightforward. Let us follow the arguments of Merton (1990) (please see also Neftci, 2000, for a treatment of Merton's arguments which we follow here) who shows that $\frac{dW}{dt}$, as is well-known, can not be defined with ordinary derivatives. This problem is circumvented in the Nelson theory by using $E\left[\frac{dW}{dt}\right]$, where *E*(*dW*) = 0.

Assume we want to impose that $\frac{dW}{dt}=0$ and we thus allow for the use of no expectation operators. We note again, we are well aware that $\frac{dW}{dt}$ does not exist. But let us do a quick thought experiment and assume it were to exist. What would be the consequences?

Define elapsed time as *h* = *t*_*k*_−*t*_*k*−1_ and let $n=\frac{T}{h^{m}}$ with *m*>1, where *T* is total time. Following Neftci (2000), assume there exists a quantity *A*_2_ so that: $\infty >A_{2}>\sum_{k=1}^{\infty }E[\Delta W_{k}^{2}]$ and there exists a quantity *A*_3_ so that $\frac{E\left[\Delta W_{k}^{2}\right]}{V_{\max}}>A_{3}$ with *A*_3_∈]0, 1[ where $V_{\max}=\underset{k}{\max}E\left[\Delta W_{k}^{2}\right]$. From the proof which allows for showing that $E\left[\Delta W_{k}^{2}\right]=\sigma_{k}^{2}h$, the following relation is essential (see Neftci, 2000): $\frac{h}{T}\frac{A_{2}}{A_{3}}>E\left[\Delta W_{k}^{2}\right]>\frac{A_{3}A_{1}}{T}h$, where $0<A_{1}<E\left[\Delta W_{k}^{2}\right]$. In order to come to show under what conditions $\frac{dW}{dt}=0$ (thus without expectation operator) we want to impose, using $n=\frac{T}{h^{m}}$ (*m*>1), that $\frac{h^{m}}{T}\frac{A_{2}}{A_{3}}>V_{\max}$. Clearly, if *h* is small (<1) then *h*^*m*^ (*m*>1) will be smaller than *h*. Hence, it is reasonable to write that: $\frac{h^{m}}{T}\frac{A_{2}}{A_{3}}<\frac{h}{T}\frac{A_{2}}{A_{3}}$. Hence, if we still want $\frac{h^{m}}{T}\frac{A_{2}}{A_{3}}>V_{\max}$, we must impose that $m>\frac{\log V_{\max}+\log\frac{A_{3}}{A_{2}}T}{\log h}$. We know that *A*_3_>0; *A*_2_>*A*_1_>0. However, *h* = *t*_*k*_−*t*_*k*−1_ must clearly be positive! We can then write that: $\frac{h^{m}}{T}\frac{A_{2}}{A_{3}}>E\left[\Delta W_{k}^{2}\right]>\frac{A_{3}A_{1}}{T}h^{m}$ and one can then define that: $E\left[\Delta W_{k}^{2}\right]=\sigma_{k}^{2}h^{m}$. We can approximate: $\frac{dW}{dt}≃\underset{h\to 0}{\lim}\frac{\Delta W}{h}=\underset{h\to 0}{\lim}\frac{h^{\frac{m}{2}}}{h}=\underset{h\to 0}{\lim}h^{\frac{m-2}{2}}$ which for *m*>2 will yield 0. Thus, we obtain that $\frac{dW}{dt}≃0$ in non expectation operator form when: (*i*) $m>\frac{\log V_{\max}+\log\frac{A_{3}}{A_{2}}T}{\log h}$ and (*ii*) *m*>2 and therefore we must impose that $\frac{\log V_{\max}+\log\frac{A_{3}}{A_{2}}T}{\log h}=2$ and this condition would mean that the uncertainty concentrates in a very specific period of time, $V_{\max}=\frac{h^{2}A_{2}}{A_{3}T}$. It is clear from the condition $\frac{\log V_{\max}+\log\frac{A_{3}}{A_{2}}T}{\log h}=2$ that there can not exist time reversibility since *h*>0 (and *h*≠1) for the log*h* to be valid. This may on prima facie, “prove” that the expectation operators which have been imposed on $\frac{dW}{dt}$ in Nelson's theory are intrinsically connected to time reversibility.

A commutativity rule such as, $E\left[x_{n}\left(\frac{x_{n+1}-x_{n}}{\epsilon }\right)-\left(\frac{x_{n}-x_{n-1}}{\epsilon }\right)x_{n}\right]\equiv E\left(C\right),$ also needs to use such operators. Without the expectation operators, setting *x*_*n*_ fixed: $x_{n}.\left[\frac{dx_{+}}{dt}-E\left(\frac{dx_{-}}{dt}\right)\right]$, is possible. Time reversibility is obtained via the expectation operator. In the set up by Nelson, a function *u*(*x, t*) is defined as: $u\left(x,t\right)=\frac{1}{2}\left(b_{+}\left(x,t\right)-b_{-}\left(x,t\right)\right)$, and this function is zero in Newtonian mechanics. We also remark in Haven (2015)^4^ that Nelson (1966) and Paul and Baschnagel (1999) define $u\left(x,t\right)=\frac{\sigma^{2}}{2}\nabla \ln\;f$, where *f* is a probability density function (for instance *f* = *e*^2*R*(*x, t*)^).

From the above, we can not define $\frac{dx_{-}}{dt}$ (since this would require time reversibility) and hence, we can not define $u\left(x,t\right)=\frac{\sigma^{2}}{2}\nabla \ln\;f$. This is the case, because the relation is obtained under time reversal on the Fokker-Planck pde: $\frac{\partial f\left(x,t\right)}{\partial t}+\nabla \left(b_{+}\left(x,t\right)f\left(x,t\right)\right)+\frac{\sigma^{2}}{2}\Delta f\left(x,t\right)=0$, which is now impossible since log*h* is to exist. If *u*(*x, t*) can not be defined then $\frac{\nabla^{2}R^{\prime }}{R^{\prime }}=\Delta R+{\left(\frac{u\left(x,t\right)}{\sigma^{2}}\right)}^{2}$ is not definable.

### 4.4. What are the real and quantum potential in lux's model?

We can write that using the real potential, *V*, $E\left[\frac{dx}{dt}\right]=\nabla V=b_{\pm }\left(x,t\right)$. In full analogy with this, we write Equation (19) now as:

(20) $$\begin{array}{l} \nabla V=\bar{x}\beta T_{c}+\beta T_{f}p_{f}-\bar{p}\beta T_{f}; \end{array}$$

which we write in shorthand format as: $\nabla V=\alpha -\bar{p}\beta T_{f}$. Recall Equation (5) which gave the expression for the “quantum potential”:

(5) $$\begin{array}{ll} \frac{\nabla^{2}R^{\prime }}{R^{\prime }}= & \frac{1}{\sigma^{2}}\frac{\partial }{\partial x}\left[\frac{1}{2}\left(b_{+}\left(x,t\right)-b_{-}\left(x,t\right)\right)\right]\\ +\frac{1}{4\sigma^{4}}\left[b_{+}{\left(x,t\right)}^{2}-2b_{+}\left(x,t\right)b_{-}\left(x,t\right)+b_{-}{\left(x,t\right)}^{2}\right]. \end{array}$$

Using Equation (20) in Equation (5), we obtain:

(21) $$\begin{array}{l} \frac{\nabla^{2}R^{\prime }}{R^{\prime }}=\frac{1}{\sigma^{2}}\frac{\partial }{\partial \bar{p}}\left[\frac{1}{2}\left(\alpha -\bar{p}\beta T_{f}\right)\right]+\frac{1}{4\sigma^{4}}\left[{\left(\alpha -\bar{p}\beta T_{f}\right)}^{2}\right]; \end{array}$$

where we have to note that $\bar{x}$ is calculated as per Lux (1997), $\underset{x}{\sum }\underset{p}{\sum }x.L\left(x,p;t\right)$; where *L*(*x, p*; *t*) is defined as the probability to occupy a state (*x, p*); i.e., where *x* is the distribution of attitudes and *p* is price and hence indirectly $\bar{x}$ is a function of *p*, via the probability *L*. Similarly, note that $\bar{p}=\underset{x}{\sum }\underset{p}{\sum }p.L\left(x,p;t\right)$. Similarly, *T*_*c*_ in Lux (1997) is defined as: *T*_*c*_≡2*Nt*_*c*_, where 2*N* is the total noise trader population and *t*_*c*_ is the amount the chartist, individually buys or sells. We assume that *t*_*c*_ is not dependent on $\bar{p}$.

Simplifying Equation (21) is now straightforward and leads to:

(22) $$\begin{array}{l} \frac{\nabla^{2}R^{\prime }}{R^{\prime }}=\frac{1}{\sigma^{2}}\frac{1}{2}\left(-\beta T_{f}\right)+\frac{1}{4\sigma^{4}}\left[{\left(\alpha -\bar{p}\beta T_{f}\right)}^{2}\right] \end{array}$$

Then multiplying Equation (22) with $\frac{-m\sigma^{4}}{2}$ and also taking $\frac{-d}{d\bar{p}}$ so as to get the force, we obtain:

(23) $$\begin{array}{l} -\nabla Q=-\frac{d}{d\bar{p}}\left[\left(\frac{-m\sigma^{4}}{2}\right)\frac{\nabla^{2}R^{\prime }}{R^{\prime }}\right]=\frac{m}{4}\left(\alpha -\bar{p}\beta T_{f}\right)\left(-\beta T_{f}\right). \end{array}$$

Recall from Equation (20), that we now can write:

(24) $$\begin{array}{l} -\nabla V=\bar{p}\beta T_{f}-\alpha . \end{array}$$

Recall that the *m* factor in −∇*Q* can indeed be very small if we move toward a quantum mechanical environment. If we thus write: −∇*V*−∇*Q*, we then obtain:

(25) $$\begin{array}{l} -\nabla V-\nabla Q=\bar{p}\beta T_{f}-\alpha +\frac{m}{4}\left(\alpha -\bar{p}\beta T_{f}\right)\left(-\beta T_{f}\right). \end{array}$$

This can be simplified to:

(26) $$\begin{array}{l} \left(\bar{p}\beta T_{f}-\alpha \right)\left[1+\beta T_{f}\left(\frac{m}{4}\right)\right]. \end{array}$$

Clearly, if *m* → 0 and σ^2^≠0 then $\beta T_{f}\left(\frac{m}{4}\right)$ is indeed very small.

We can also write: $\frac{\nabla V}{\nabla R}=2\sigma^{2}$ and hence: $\nabla R=\frac{\nabla V}{2\sigma^{2}}$. We can then write that:

(27) $$\begin{array}{l} R\left(\bar{p}\right)=\frac{1}{2\sigma^{2}}\int \alpha -\bar{p}\beta T_{f}d\bar{p}; \end{array}$$

which is worked out as: $R\left(\bar{p}\right)=\frac{1}{2\sigma^{2}}\left(\alpha \bar{p}\right)-\frac{1}{4\sigma^{2}}\beta T_{f}{\bar{p}}^{2}+C.$ Recall that $s=\frac{1}{1+r}\int \exp\left(2R\left(\bar{p},t\right)\right)d\bar{p}$, where *s* is the state price. In the context of the model we consider here this would yield:

(28) $$\begin{array}{l} s=\frac{1}{1+r}\int \exp\left(\left(\frac{1}{\sigma^{2}}\left(\alpha \bar{p}\right)-\frac{1}{2\sigma^{2}}\beta T_{f}{\bar{p}}^{2}+C\right)\right)d\bar{p}. \end{array}$$

This means that the state price (an insurance price which is paid to guarantee a financial outcome when a particular state of nature occurs) is now dependent, using Lux's model which is embedded in our quantum/real potential model, on the (i) volatility; (ii) the expected price of an asset (which chartists and fundamental buyers buy); (iii) the parameter β for the average speed of price adjustment in the presence of excess demand; (iv) the term $\alpha =\bar{x}\beta T_{c}+\beta T_{f}p_{f}$. Remark that this density function used the amplitude function *R* from $\nabla R=\frac{\nabla V}{2\sigma^{2}}$. The quantum potential in its full form is absent from this relation, but parts of that potential (i.e., ∇*R*) are still figuring in the equation. In this formulation the preference factor (via $\bar{x}$) would be embedded in $\bar{p}$. The volatility parameter seems to be the “conduit” factor which links the changes in *R* with the changes in *V*, via ∇*V* = 2σ^2^∇*R*. Thus, Equation (28) does only exist here if this “conduit” factor exists (i.e., if σ^2^is not zero).

## 5. What is the relevance of real and quantum potentials in empirical work?

This paper has not yet answered a crucial question, for which we have to thank one of the referees of this paper. Can the quantum and/or real potentials have empirical relevance? The answer is fortunately enough positive. Recent work by Belal Baaquie shows quite clearly how real potential functions can be estimated for traded commodities. Please consult Baaquie (2013). The same author also shows how the minimization of a real potential function, when that function is defined as being equal to the sum of supply and demand functions, yields a more general version of the equilibrium price which is well-known in basic economics. The quantum potential can also be estimated from real market data. In the paper by Tahmasebi et al. (2015) the quantum potential is estimated for the Standards and Poor (S&P) Index. More work is needed on how the path derived from the extended Newtonian motion (i.e., with thus two potentials), can be used to emulate price behavior over time.

## 6. Conclusion

We have attempted to show that preferences for risk seem to be captured by both types of potentials (i.e., the real and quantum potentials). When embedding the basics of the Lux model in the types of potential approach proposed in this paper, we seem to find that the quantum potential's influence depends on the mass parameter. This parameter varies depending on whether we are far removed (or not) from the quantum physical limit. The last section of the paper does indicate that the real and quantum potentials can be estimated within real financial data settings. But the question may remain, if the proposed analysis in this paper is of any value, how one can interpret the quantum potential in light of those real data interpretations? In the paper by Tahmasebi et al. (2015) it is shown quite clearly that a quantum potential with infinite walls occurs for the S&P Index for short time scales, and when the time scale grows those infinite walls disappear and the quantum potential for the S&P index for large time scales resembles the quantum potential for Gaussian white noise. Price variation is deemed to be very small for small time scales, but allowed to be larger for large time scales. This is intuitively acceptable.

From a Newtonian price path point of view, considering for instance Equation (25), our analysis in this paper seems to indicate, that the influence of the quantum potential (next to the real potential) on the price path, may vary. But to pinpoint, in an economics sense, what this parameter of variation really means is very difficult.

### Conflict of interest statement

The author declares that the research was conducted in the absence of any commercial or financial relationships that could be construed as a potential conflict of interest.

## Footnotes

^1^This is Equation (25) in that paper.

^2^This is Equation (26).

^3^This is Equation (36).

^4^This is Equation (19).

## References

[B1] Baaquie B. (2013). Statistical microeconomics. Physica A 392, 4400–4416.

[B2] Haven E. Khrennikov A. (2013). Quantum Social Science. New York, NY: Cambridge University Press.

[B3] Haven E. (2015). Potential functions and the characterization of economics-based information. Found. Phys. 45, 1394–1406. 10.1007/s10701-015-9931-4

[B4] Khrennikov A. Y. (1999). Classical and quantum mechanics on information spaces with applications to cognitive, psychological, social and anomalous phenomena. Found. Phys. 29, 1065–1098.

[B5] Lux T. (1997). Time variation of second moments from a noise trader infection model. J. Econ. Dyn. Control 22, 1–38.

[B6] Merton R. (1990). Continuous Time Finance. Oxford: BlackwellPress.

[B7] Neftci S. (2000). An Introduction to the Mathematics of Financial Derivatives. New York, NY: AcademicPress.

[B8] Nelson E. (1966). Derivation of the Schrödinger equation from newtonian mechanics. Phys. Rev. 150, 1079–1085.

[B9] Nelson E. (1985). Quantum Fluctuations. Princeton, NJ: Princeton University Press.

[B10] Paul W. Baschnagel J. (1999). Stochastic Processes: From Physics to Finance. Heidelberg: Springer Verlag.

[B11] Tahmasebi F. Meskinimood S. Namaki A. Vasheghani Farahani S. Jalalzadeh S. Jafar G. R. (2015). Financial market images: a practical approach owing to the secret quantum potential. Eur. Lett. 10.1209/0295-5075/109/30001

